# Longitudinal and Gender Measurement Invariance of the General Health Questionnaire-12 (GHQ-12) From Adolescence to Emerging Adulthood

**DOI:** 10.1177/10731911241229573

**Published:** 2024-02-12

**Authors:** Pascal Schlechter, Sharon A. S. Neufeld

**Affiliations:** 1 Department of Psychiatry, University of Cambridge, UK

**Keywords:** psychological distress, GHQ-12, measurement invariance, adolescents, emerging adulthood

## Abstract

Psychological distress often onsets during adolescence, necessitating an accurate understanding of its development. Assessing change in distress is based on the seldom examined premise of longitudinal measurement invariance (MI). Thus, we used three waves of data from Next Steps, a representative cohort of young people in the UK (*N* = 13,539) to examine MI of the General Health Questionnaire-12 (GHQ-12). We examined MI across time and gender from ages 15 to 25 in four competing latent models: (a) a single-factor model, (b) a three-factor correlated model, (c) a bifactor model of “general distress” and two orthogonal specific factors capturing positive and negative wording, and (d) a single-factor model including error covariances of negatively phrased items. We also tested acceptability of assumptions underlying sum score models. For all factor models, residual MI was confirmed from ages 15 to 25 years and across gender. The bifactor model had the best fit. While sum score model fit was not unequivocally acceptable, most mean differences across time and gender were equivalent across sum scores and latent difference scores. Thus, GHQ-12 sum scores may be used to assess change in psychological distress in young people. However, latent scores appear more accurate, and model fit can be improved by accounting for item wording.

Mental health problems often onset during early adolescence (Solmi et al., 2022) and present a leading cause of disease burden among adolescents and emerging adults (Armocida et al., 2022). For prevention purposes, adolescence to emerging adulthood thus constitutes a critical period to understand unfolding psychological distress (Dahl et al., 2018). Psychological distress is commonly estimated with the General Health Questionnaire (GHQ), and meta-analyses suggest a high prevalence of psychological distress in adolescents when using the GHQ-12 (Silva et al., 2020). The GHQ-12 assesses general psychological distress via symptoms of common mental health problems including anxiety, depression, somatic symptoms, and social dysfunction (Gnambs & Staufenbiel, 2018). However, developmental processes and the emergence of gender differences complicate an accurate estimation of the prevalence of psychological distress across time. Nonetheless, many applied studies use GHQ-12 sum scores to quantify general symptom load (Tseliou et al., 2018). However, this approach is oversimplified and can obfuscate precise symptom assessment, which is necessary for adequate resource allocation for intervention and prevention (Breedvelt et al., 2020; McNeish, 2022). To date, it remains unknown whether the GHQ-12 measures the same construct from adolescence to emerging adulthood across time and gender, and whether sum score models derived from the GHQ-12 can adequately inform epidemiological research. The present study addresses these gaps by investigating temporal measurement invariance (MI) of different factor solutions that have been proposed for the GHQ-12 from age 15 to 25 and across gender.

## The General Health Questionnaire-12

The GHQ-12 is a very commonly used instrument in epidemiological research assessing mental distress in the past 2 weeks by using a four-point Likert-type scale (Gnambs & Staufenbiel, 2018). Multiple versions of the GHQ with different numbers of items exist (Goldberg et al., 1997). In practice, the GHQ-12 is particularly advantageous for applied research due to its brevity and applicability as a screening instrument (Böhnke & Croudace, 2016). Given its good reliability and validity, the GHQ-12 is widely used in clinical practice (Richardson et al., 2007) and epidemiological research (Henkel et al., 2003) to index psychological distress. However, ongoing debates surround the dimensionality of GHQ-12, which is intended to be unidimensional (Goldberg & Williams, 2000). Some studies have reported two- or three-factor models based on exploratory factor analyses (Gelaye et al., 2015; Guan, 2017). However, the two-factor solution mainly reflects method effects of positively and negatively phrased items instead of capturing distinct meaningful factors (Hankins, 2008; Rey et al., 2014). The most prominent three-factor model consists of the factors social dysfunction, anxiety/depression, and loss of confidence and was found in adults and adolescents (Abubakar & Fischer, 2012; French & Tait, 2004; Graetz, 1991). However, the three-factor model captures all negative items in the anxiety/depression factor and confidence items while the social dysfunction factor contains all items worded positively. Therefore, depending on the factor model, the covariation among item subsets is either attributable to method effects (e.g., negative wording) or substantial factors (e.g., reflecting a distinct social dysfunction factor). Meta-analyses suggest that the GHQ-12 essentially reflects a one-dimensional construct with other factors (mostly method factors) explaining little meaningful variance (Gnambs & Staufenbiel, 2018). Moreover, a recent analysis of adolescents aged 14–16 indicated that the GHQ-12 items reflect a unidimensional construct (Pérez et al., 2020). To account for the shared variance of positively and negatively phrased items, studies have either used a bifactor approach with one overarching general distress factor and two orthogonal latent method factors (Hystad & Johnsen, 2020) or allowed for the covariances of errors between negatively phrased items (King et al., 2023). However, in line with our aforementioned arguments, the two specific factors in the bifactor model could also represent social dysfunction and anxiety/depression/confidence-specific factors.

**Figure 1.**
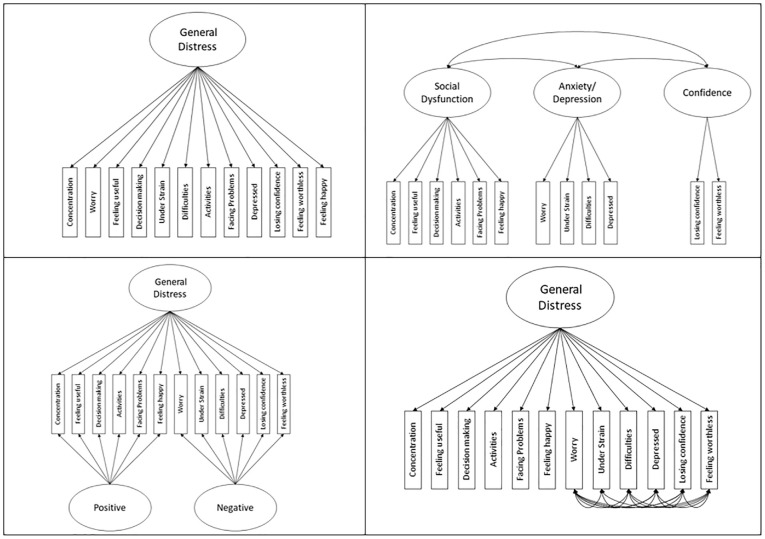
Schematic Representation of the Three Different Factor Solutions: One-Factor Solution (Upper Left Panel, Model 1), Three-Factor Solution (Upper Right Panel, Model 2), Bifactor Solution (Lower Left Panel, Model 3), and a One-Factor Solution With Correlated Errors of Negatively Phrased Items (Lower Right Panel, Model 4).

To accommodate findings in the existing literature, we tested MI of the GHQ-12 across time and gender in four models that are visually depicted in Figure 1. Model 1 presents a simple one-factor solution with one latent general distress factor. Model 2 is a three-factor solution with correlated latent factors (“Social Dysfunction,”“Anxiety/Depression,”“Confidence”). Model 3 is a bifactor model with one overarching latent “general distress” trait and two orthogonal latent specific factors capturing the unique variance attributable to the positive and negative item wording. Model 4 reflects a one-dimensional factor allowing for covariance of error terms of the negatively phrased items.

## Measurement Invariance

While epidemiological research indicates an increase in mental health problems during adolescence that pervade into emerging adulthood (Solmi et al., 2022), these observations are often derived from total scale scores that assume one underlying latent construct (Silva et al., 2020). However, for these conclusions to be valid, it needs to be established whether these manifested mean differences reflect true-score differences in the latent construct (Olino, 2020). Likewise, gender differences in psychological distress which start to unfold during early adolescence (Patalay & Fitzsimons, 2018; Shore et al., 2018; Solmi et al., 2022) need to be truly attributable to differences in the latent construct, and not measurement differences, in order to derive meaningful clinical implications (Liu et al., 2017). Given the multitude of social, psychological, and biological changes throughout adolescence and emerging adulthood (Dahl et al., 2018), it could be that items tapping into psychological distress have a different meaning for young people across development. Several assumptions need to be tested first before differences in covariances or means across time or gender can be truly attributed to differences in the latent construct (Liu et al., 2017). In a first step, it is important to investigate whether the factor structure is equivalent across time or gender (configural MI). When the same factor structure is established across time or gender, factor loadings are additionally constrained to be equal across measurement occasions or gender to test whether the symptoms relate to the latent construct in the same way over time or across gender (weak/metric MI). Then, item thresholds are additionally constrained to be equivalent to examine whether the observed thresholds conditional on the latent factor do not differ across time or gender (strong/scalar MI). Last, residual variances of the items are additionally constrained to be equal to gauge whether the amount of variance in the items not accounted for by the latent factor is the same across time or gender (residual/strict/unique factor MI). In a further step, one can test the strict assumption of sum score models by setting all factor loadings within one factor equal. Even if longitudinal MI is supported, items still contribute differently to the underlying construct (i.e., having different factor loadings; McNeish, 2022; McNeish & Wolf, 2020; Widaman & Revelle, 2023). Thus, treating items equally in composite scores may lead to individuals having the same manifest score while their relative standing on the latent trait differs. This may obscure the detection of true differences between individuals and can result in different conclusions based on these scores if their assumptions remain untested (McNeish & Wolf, 2020; Widaman & Revelle, 2023).

MI of different GHQ-12 factor models has been tested in adults across various scenarios: clinical and non-clinical populations (Fernandes & Vasconcelos-Raposo, 2013), different ethnic groups (Bowe, 2017; King et al., 2023), different cultures (Romppel et al., 2017), gender (Shevlin & Adamson, 2005), time (Mäkikangas et al., 2006), and before and during COVID-19 (Schlechter, 2023). However, MI of the GHQ-12 across time and gender as well as the assumptions of sum score models remain untested for the developmental period from adolescence to emerging adulthood.

## Developmental Changes

As young people are undergoing developmental transitions including a changing social network, brain maturation, or hormonal changes, this could lead to a different representation of the construct of psychological distress across time (Dahl et al., 2018). Specifically, symptoms may change in their underlying meaning across development. Having concentration problems may not be reflective of psychological distress at earlier ages but rather signify challenges of staying focused in school or other problems like attention or hyperactivity (Meinzer et al., 2014). Indeed, in a MI analysis of a measure of depression in young people, the item “hard to concentrate” had relatively low factor loadings on the depression construct across development, especially at ages 11 and 13 (Schlechter et al., 2023). In addition, self-concept is changing across adolescence (Coughlin & Robins, 2017), and thus the mental representation of the items “feeling worthless” and “losing confidence” and their connection with psychological distress may change accordingly. This may be reflected in lower factor loadings on these items at younger ages if, for instance, adults have a more stable lack of confidence that is more strongly linked to psychological distress. Likewise, playing a useful role and feeling capable to make decisions may be differently related to the distress latent construct over development. For example, younger adolescents are more dependent on others than emerging adults and may feel less able to play a useful role or feel capable of making decisions (Dahl et al., 2018). Therefore, these symptoms may be more weakly linked to psychological distress in adolescents than in emerging adults. In sum, it needs to be established whether GHQ-12 items are equally reflective of psychological distress during a life phase that is characterized by physical, emotional, and social change (Dahl et al., 2018). Only when measurement properties do not deviate from each other over time can manifest mean differences serve as an approximation of change in psychological distress (Liu et al., 2017).

## Gender Differences

An accurate assessment of psychological distress across development becomes even more challenging as gender differences in psychological distress start to unfold during early adolescence (Patalay & Fitzsimons, 2018; Shore et al., 2018; Solmi et al., 2022). For instance, women report higher rates of major depression than men, with a ratio around 2:1 (Hyde & Mezulis, 2020; for a meta-analysis, see Salk et al., 2017) and display an earlier and steeper increase in depressive symptoms than males during the ages of 12–15 years (Patalay & Fitzsimons, 2018; Shore et al., 2018; Solmi et al., 2022). Differences in psychological distress could arise from a variety of social (e.g., gender roles; Anyan & Hjemdal, 2018), biological (e.g., pubertal development; Lewis et al., 2018), and psychological factors (e.g., differences in rumination tendencies; Nolen-Hoeksema & Aldao, 2011). However, higher endorsement of certain items may also reflect different psychological or biological processes, or social norms in the way that females and males experience or express different symptoms.

Social aspects may play an important role in this regard. From early adolescence to emerging adulthood, individuals grow into social roles that shape the behavior and the expression of feelings in various contexts (Anyan & Hjemdal, 2018). Men may be socialized to be strong and may be less likely to express negative feelings (Anyan & Hjemdal, 2018). In females, mood-related symptoms may be perceived as part of their normative experiences, which also could vary with hormonal fluctuations (Hyde et al., 2008; Payne, 2003). This may be reflected in different item thresholds across gender, for example, with females being more likely to endorse feeling unhappy at lower levels on the distress latent construct.

Biological differences between sexes are also important to consider. Puberty and the accompanying hormonal changes have been found to contribute to the manifestation of psychological distress differently in females and males (Lewis et al., 2018). The interplay between these hormonal changes and environmental demands may result in a higher incidence of self-reported depressed mood or feelings of worthlessness in females than in males (Hyde et al., 2008), resulting in greater endorsement of the depressed and feeling worthless items in females. Testing whether these items reflect psychological distress equally across gender in different developmental stages is thus warranted. Only if the construct of psychological distress is equally represented across gender do mean-level differences reflect true-score differences (Hyde & Mezulis, 2020; Salk et al., 2017).

## The Present Study

In the present study, we used data from Next Steps, a national cohort study representative of the United Kingdom (UK) to systematically examine MI of the GHQ-12 across time and gender from adolescence into emerging adulthood. Based on prior literature, we examined temporal MI from age 15 to 25 and MI across gender for each measurement occasion in a simple one-factor solution (Model 1), a three-factor solution with correlated latent factors (Model 2), a bifactor model with two specific factors capturing the unique variance attributable to the positive and negative item wording (Model 3), and a one-dimensional factor allowing for the covariance of the error terms of the negatively phrased items (Model 4, see Figure 1). In addition, we tested whether sum score models serve as an adequate representation of the data, and whether manifest mean differences across time and gender differ from latent mean differences of MI models where the highest level of MI is modeled.

## Method

### Next Steps Cohort Study

Next Steps is a national cohort study representative of young people in the UK (formerly known as Longitudinal Study of Young People in England). Cohort members were born between September 1, 1989, and August 31, 1990. They were initially recruited in schools around the age of 14. The population consists of participants enrolled in Year 9 in English state and independent schools and pupil referral units in 2004. Deprived schools and ethnic minorities were oversampled. The initial sample at baseline comprised approximately 21,000 young people. Of those, 15,770 young people were interviewed at baseline. At age 17, an additional minority sample was added. Eight waves of data collection took place, with the most recent wave at age 25. Gender was assessed by a single question that did not differentiate between biological sex and gender identity. More information about the Next Steps study has been described elsewhere (Calderwood & Sanchez, 2016). Ethical approval for the study was given by the NHS Research Ethnics Committee. Table 1 shows demographic characteristics of the sample. Data are openly available through the UK data service. This study’s design and its analysis were not preregistered.

**Table 1:** Demographic Characteristics Per Wave

Demographic characteristics	Age 14–15 (*N* = 13,539)	Age 16–17 (*N* = 11,801)	Age 25–26 (*N* = 6,792)
Age	14.67 (*SD* = 0.47)	16.68 (*SD* = 0.48)	25.28 (*SD* = 0.44)
Gender			
Male	6,740 (50.6%)	5,832 (50.3%)	2,954 (43.5%)
Female	6,577 (49.3%)	5,765 (49.7%)	3,712 (54.7)
Ethnicity			
White	9,133 (68.7%)	7,973 (67.7%)	4,950 (72.9)
Mixed	670 (5.0%)	592 (5.0%)	84 (1.2%)
Indian	830 (6.2%)	767 (6.5%)	468 (6.8%)
Pakistani	831 (6.2%)	669 (5.7%)	375 (5.5%)
Bangladeshi	623 (4.7%)	524 (4.5%)	305 (4.5%)
Black Caribbean	453 (3.4%)	470 (3.9%)	200 (2.9%)
Black African	445 (3.3%)	485 (4.1%)	174 (2.6%)
Other	313 (2.4%)	303 (2.6%)	134 (2.0%)
Employment MP			
Full-time paid employee	4,789 (35.4%)	5,151 (43.6%)	—
Part-time paid employee	3,638 (26.8%)	2,980 (25.3%)	—
Full-time self-employed	471 (3.5%)	—^a^	—
Part-time self-employed	209 (1.5%)	—^a^	—
Unemployed/seeking work	303 (2.2%)	302 (2.5%)	—
Full-time education	119 (1.0%)	85 (1.0%)	—
Employment training	17 (0.1%)	22 (0.1%)	—
Temporarily sick/disabled	110 (1.0%)	69 (1.0%)	—
Permanently sick/disabled	349 (2.6%)	362 (3.1%)	—
Looking after home/family	3,020 (22.3%)	2,197 (18.6%)	—
Retired from work	145 (1.0%)	184 (1.5%)	—
Other	38 (0.2%)	46 (0.3%)	—

*Note.* At age 25, parents were not interviewed. MP = main parent.

a At age 17, these response categories were not assessed.

### General Health Questionnaire-12

The GHQ-12 is a 12-item self-report questionnaire assessing mental distress in the past 2 weeks by using a four-point Likert-type scale (Goldberg & Williams, 2000). In the Next Steps study, GHQ was assessed at ages 15, 17, and 25 years. Across general adult populations, the GHQ-12 is well validated (Gnambs & Staufenbiel, 2018).

### Data Analysis

#### Missingness

Analyses were performed with R Version 4.0.3 (R Core Team, 2021). Levels of missingness across waves were high (Table 1). At age 15, *N* = 13,539 young people participated, while at age 25, only *N* = 6,792 participated (50.5% compared to that at age 15). Levels of missingness were associated with male gender, maternal education, and employment, as well as ethnicity (Supplemental Table S1). Given the presence of predictors of missingness and in line with previous missingness analyses on this sample (Silverwood et al., 2020), we assumed that data were missing at random (MAR) (White et al., 2011). As our data were ordinal, we applied the weighted least squares mean and variance adjusted (WLSMV) estimator for the estimation of all models (Asparouhov & Muthén, 2010). Simulation studies of MI testing suggest that the WLSMV estimator produces relatively unbiased parameters and standard error estimates even with 50% MAR missingness when the sample size is 1,000 or more (Chen et al., 2020). Nonetheless, as a sensitivity analysis, we estimated the same models using multiple imputation. Data were imputed with the MICE package in R (van Buuren et al., 2015) using proportional odds (White et al., 2011). In our imputation models, demographic characteristics that were associated with missingness were included as auxiliary variables. Ten imputed datasets were produced using demographic variables that were associated with missingness as auxiliary variables in our imputation model. This number of imputations was chosen as it enables precise point estimates while reducing computational demands (White et al., 2011), and accurate standard errors were not required for the present MI analyses.

#### Tested Models

In line with previous literature, we tested the four different models that are visually depicted in Figure 1 and have been described in the introduction.

#### MI Across Time and Gender

We ran a series of models to test MI across time and gender. For longitudinal invariance, models were tested across the three assessments at ages 15, 17, and 25. In all longitudinal models, the covariances between errors of the same indicators were allowed over time. In addition, all factors were allowed to covary over time expect for the bifactor model where only the same factors (e.g., overarching distress factor) were allowed to covary over time. For gender, invariance constraints were placed on three separate cross-sectional models. Note that our gender MI analysis only refers to a single question and is not able to provide a nuanced differentiation between biological sex and gender. MI of all factor solutions across time and gender was tested by comparing increasingly constrained models in a confirmatory factor analysis (CFA) framework (Liu et al., 2017), following the steps outlined in the introduction. That is, we tested the assumptions of configural, metric, scalar, and residual MI as outlined by Millsap and Yun-Tein (2004). To test constraints on the residual variances, we used theta parameterization in our models (Edossa et al., 2018). In all models, the Comparative Fit Index (CFI) should be above .95 (.90 for acceptable fit) and the Root Mean Square Error of Approximation (RMSEA) below .05 (below .08 for acceptable fit) to index good model fit (Hu & Bentler, 1999). Differences in the χ^2^ test statistic were not investigated because they are likely significant given our large sample size (Liu et al., 2017). To test for each level of MI, changes in the fit indices for each model were compared to the previously established level of MI. In line with recent recommendations for MI on bifactor models, ΔCFI ≥ .010 and ΔRMSEA ≥ .007 indicate substantial deterioration in model fit (Neufeld et al., 2023).

#### Sum Score Model Testing

For the one- and three-factor solutions, we tested whether sum score model constraints adequately fit the data. To this end, all factor loadings within each wave for each latent factor were set to be equal. We compared model fit to the unconstrained configural model using the same model comparison criteria as for MI testing (McNeish & Wolf, 2020; Widaman & Revelle, 2023).

#### Standardized Mean Differences

We tested potential consequences of using unweighted sum scores compared to analyzing latent mean differences. To establish this, we calculated standardized GHQ-12 differences of the sum scores across the three measurement waves and compared them to the standardized latent mean differences across time for the unidimensional model (Model 1), the general latent factor of the bifactor model (Model 3), and the correlated error model (Model 4). The same comparisons were made for sum scores of the three subscales (social dysfunction, anxiety/ depression, and confidence) versus the latent scores of the three-factor solution (Model 2). Finally, we compared the manifest sum score mean differences across gender to latent mean differences across gender for all models. For the latent mean differences, the model with the highest established level of MI was used. To compare the estimates resulting from the different scoring methods, we report 99% confidence intervals around them.

## Results

### Descriptive Statistics

Descriptive statistics of single items and scale composite scores over time are presented in Table 2. The internal consistencies were good for age 15 (α = .86; $\omega_{t}$ = .86), age 17 (α = .86; $\omega_{t}$ = .86), and age 25 (α = .89; $\omega_{t}$ = .90). Table 3 depicts freely estimated factor loadings for the different models. For the one- and three-factor solution, factor loadings were good (all λ’s > .30). Likewise, factor loadings on the general factor and the specific factor capturing unique variance of the positively phrased items in the bifactor model were good. However, two-factor loadings on the specific factor capturing unique variance of the negatively phrased items were poor (i.e., losing confidence and feeling depressed had loadings below .2). In addition, the items losing confidence and feeling worthless had negative factor loadings on the specific factor. In Supplemental Tables S2 and S3, we report analysis using multiple imputation. All MI results were confirmed in this sensitivity analysis.

**Table 2:** Descriptive Statistics of the General Health Questionnaire-12 Across Waves

Age	15				17				25			
Items	*M*	*SD*	*SK*	*KT*	*M*	*SD*	*SK*	*KT*	*M*	*SD*	*SK*	*KT*
Been able to concentrate on what you’re doing?	2.01	0.62	0.66	1.69	2.01	0.62	0.53	1.26	2.08	0.65	0.61	1.13
Lost much sleep over worry?	1.77	0.89	0.95	0.05	1.92	0.92	0.69	−0.44	2.07	0.84	0.54	−0.18
Felt you were playing a useful part in things?	1.92	0.61	0.84	2.67	1.91	0.65	0.72	1.67	2.01	0.67	0.71	1.34
Felt capable of making decisions about things?	1.77	0.60	0.56	1.54	1.70	0.64	0.67	0.87	1.87	0.60	0.55	1.68
Felt constantly under strain?	1.97	0.94	0.66	−0.53	2.14	0.95	0.38	−0.82	2.27	0.83	0.36	−0.34
Felt you couldn’t overcome your difficulties?	1.85	0.89	0.87	−0.01	1.88	0.88	0.75	−0.22	1.95	0.80	0.66	0.13
Been able to enjoy your normal day-to-day activities?	1.90	0.65	0.72	1.69	1.95	0.70	0.62	0.75	2.12	0.63	0.78	1.59
Been able to face up to your problems?	1.83	0.65	0.75	1.66	1.86	0.65	0.65	1.38	1.99	0.60	0.71	2.21
Been feeling unhappy and depressed?	1.88	0.96	0.83	−0.36	1.90	0.98	0.73	−0.62	1.98	0.90	0.62	−0.41
Been losing confidence in yourself?	1.70	0.91	1.14	0.31	1.69	0.91	1.12	0.21	1.89	0.90	0.75	−0.28
Been thinking of yourself as a worthless person?	1.49	0.83	1.69	2.01	1.45	0.80	1.79	2.33	1.50	0.80	1.57	1.70
Been feeling reasonably happy, all things considered?	1.87	0.69	0.78	1.35	1.90	0.68	0.62	0.91	1.97	0.67	0.67	1.20
Total score mean	1.82	.048	1.20	0.85	1.85	0.49	1.10	1.32	1.97	0.51	1.21	1.78
Three factors												
Social dysfunction	1.87	0.41	0.63	2.53	1.89	0.42	0.72	1.88	2.01	0.45	1.01	2.79
Anxiety/Depression	1.86	0.73	0.80	0.05	1.96	0.74	0.61	−0.29	2.06	0.68	0.66	0.20
Confidence	1.59	0.80	1.44	1.39	1.57	0.77	1.46	1.47	1.70	0.77	1.19	0.85

*Note.* The stem for the items is: *“Have you recently . . .”. SK* = skewness; *KT* = kurtosis

**Table 3:** Freely Estimated Standardized Factor Loadings for All Factor Solutions

Item	Age 15							Age 17							Age 25						
	Model 1	Model 2			Model 3			Model 1	Model 2			Model 3			Model 1	Model 2			Model 3		
	1F	Soc	Dep/anx	Conf	G	Pos	Neg	1F	Soc	Dep/anx	Conf	G	Pos	Neg	1F	Soc	Dep/anx	Conf	G	Pos	Neg
Concentration	.56	.68	—	—	.48	.40	—	.55	.64	—	—	.48	.37	—	.64	.70	—	—	.54	.43	—
Lost sleep	.68	—	.71	—	.68	—	.27	.69	—	.73	—	.67	—	.35	.69	—	.72	—	.67	—	.32
Useful	.44	.54	—	—	.32	.51	—	.47	.56	—	—	.37	.48	—	.62	.69	—	—	.51	.49	—
Decisions	.39	.50	—	—	.25	.58	—	.45	.53	—	—	.31	.62	—	.61	.67	—	—	.45	.64	—
Under strain	.71	—	.73	—	.70	—	.46	.69	—	.71	—	.66	—	.52	.69	—	.72	—	.67	—	.57
Difficulties	.74	—	.77	—	.74	—	.22	.73	—	.76	—	.72	—	.20	.75	—	.79	—	.76	—	.17
Enjoy	.56	.68	—	—	.44	.52	—	.55	.64	—	—	.49	.32	—	.69	.75	—	—	.61	.38	—
Problems	.50	.61	—	—	.38	.54	—	.54	.64	—	—	.43	.56	—	.56	.62	—	—	.46	.47	—
Depressed	.84	—	.88	—	.86	—	.04	.85	—	.90	—	.87	—	.10	.86	—	.91	—	.88	—	.06
Confidence	.85	—	—	.91	.87	—	−.17	.84	—	—	.89	.87	—	−.15	.84	—	—	.90	.87	—	−.09
Worthless	.83	—	—	.88	.86	—	−.22	.83	—	—	.88	.86	—	−.20	.85	—	—	.90	.90	—	−.21
Happy	.63	.76	—	—	.55	.42	—	.65	.76	—	—	.59	.38	—	.73	.81	—	—	.67	.37	—

*Note.* Factor loadings for Model 4 are not depicted as they mirror the factor loadings of Model 1. 1F = one factor; Soc = social dysfunction factor; Dep/anx = Depression/Anxiety factor; Conf = confidence factor; G = general factor; Pos = positively worded items specific factor; Neg = negatively worded items specific factor.

### MI Across Time and Testing Assumptions of Sum Score Models

#### One-Factor Solution (Model 1)

Model fit for the one-factor solution was good according to the CFI and acceptable according the RMSEA. Over time, correlations between latent factors ranged from .26 (age 15, age 25) to .46 (age 15, age 17). Across all levels of MI, model fit did not deteriorate according to our criteria, thus confirming residual invariance for this one-dimensional factor solution. However, the model based on sum score assumptions (equal factor loadings) showed a substantial decrement in the fit indices compared with the configural model. Moreover, sum score model fit was only acceptable according to the CFI, but not according to the RMSEA (see Table 4).

**Table 4:** Longitudinal MI Models

Model	One factor (Model 1)					Three factors (Model 2)				
MI level	$\chi^{2}(\mathrm{df})$	CFI	RMSEA	ΔCFI	ΔRMSEA	$\chi^{2}(\mathrm{df})$	CFI	RMSEA	ΔCFI	ΔRMSEA
Configural	11,400 (555)	.969	.065			3,982 (522)	.990	.038		
Metric	12,431 (577)	.966	.067	.003	.002	5,055 (540)	.987	.043	.003	.005
Scalar	14,686 (613)	.959	.070	.007	.003	6,500 (576)	.983	.047	.004	.004
Strict	15,387 (649)	.957	.070	.002	.000	7,944 (612)	.979	.049	.004	.002
Sum	30,951 (588)	.912	.106	**.057**	**.041**	8,280 (549)	.978	.055	**.012**	**.017**
Model	Bifactor (Model 3)					Correlated errors (Model 4)				
Configural	2,309 (513)	.995	.028			7,792 (510)	.979	.056		
Metric	3,261 (555)	.992	.032	.003	.004	8,492 (532)	.977	.057	.002	.001
Scalar	5,686 (591)	.985	.043	.007	**.011**	10,772 (568)	.971	.062	.006	.005
Strict	6,228 (627)	.984	.040	.001	.003	11,466 (604)	.969	.062	.002	.000

*Note.*ΔCFI ≥ .010 and ΔRMSEA ≥ .007 indicate substantial deterioration in model fit (Neufeld et al., 2023). Models are compared with the prior model consisting of one less level of constraints. Bolded values indicate a substantial decrement in model fit. MI = measurement invariance; CFI = Comparative Fit Index; RMSEA = Root Mean Square Error of Approximation; *df* = degrees of freedom.

#### Three-Factor Solution (Model 2)

Model fit was good according to both fit indices. Associations among latent factors over time were between .17 (social factor: age 15, anxiety/depression factor: age 25) and .46 (anxiety/depression factor: age 15, anxiety/depression factor: age 17). In addition, we could establish residual MI across time. The sum score model had good model fit according to the CFI and acceptable model fit according to the RMSEA. However, we observed a substantial decrement in the model fit for the sum score model according to both indices (see Table 4).

#### Bifactor Model (Model 3)

Model fit was good according to both fit indices. Indeed, the bifactor model had the best descriptive model fit of all tested factor solutions. The associations among latent factors ranged from .10 (positive wording factor: age 15, positive wording factor: age 25) to .51 (general factor: age 15, general factor: age 17). ΔCFI supported residual MI, while ΔRMSEA indicated deterioration in model fit when transitioning from the metric invariance model to the scalar invariance model (see Table 4). Several item thresholds were lower for age 25 than for the other ages, indicating that response options have been endorsed at lower trait levels at this age than at ages 15 and 17. This was the case for most items, but particularly for “lost sleep” and “feeling under strain.” According to omega hierarchical, the overarching factor captured 69% of the variance at ages 15 and 17, and 75% of the variance at age 25, with non-overlapping 95% confidence intervals for age 25 compared with ages 15 and 17, respectively.

#### Correlated Errors (Model 4)

The one-dimensional model that allowed for error covariances among the negatively phrased items showed good model fit according to the CFI and acceptable model fit according to the RMSEA. Over time, correlations between latent factors ranged from .29 (age 15, age 25) to .54 (age 15, age 17). This model displayed residual MI (see Table 4).

### MI Across Gender

#### One-Factor Solution (Model 1)

Separately at all three ages (15, 17, and 25), model fit was good according to the CFI but not acceptable according to the RMSEA. As sensitivity check, we tested the models separately for males and females. The results remained the same, as CFI indicated good model fit in both cases, but the RMSEA did not. However, when putting the gender invariance constraints on the models, we did not detect substantial deterioration in model fit in any of the tested models (see Table 5).

**Table 5:** Measurement Invariance Across Gender

Age	$\chi^{2}(\mathrm{df})$	CFI	RMSEA	ΔCFI	ΔRMSEA
One-factor solution (Model 1)					
Age 15					
Configural	8,196 (108)	.956	.122		
Metric	8,537 (119)	.954	.118	.002	.004
Scalar	8,486 (142)	.955	.108	.001	**.010**
Strict	8,714 (154)	.953	.105	.002	.003
Age 17					
Configural	6,186 (108)	.963	.105		
Metric	6,262 (119)	.962	.100	.001	.005
Scalar	6,450 (142)	.961	.093	.001	.**007**
Strict	6,531 (154)	.961	.090	.000	.003
Age 25					
Configural	4,377 (108)	.978	.109		
Metric	4,476 (119)	.978	.107	.000	.002
Scalar	4,597 (142)	.977	.097	.001	**.010**
Strict	4,759 (154)	.977	.095	.000	.002
Three-factor solution (Model 2)					
Age 15					
Configural	2,229 (102)	.988	.064		
Metric	2,394 (111)	.988	.064	.000	.000
Scalar	2,460 (132)	.987	.059	.001	.005
Strict	2,599 (144)	.987	.058	.000	.001
Age 17					
Configural	2,336 (102)	.986	.065		
Metric	2,375 (111)	.986	.063	.000	.002
Scalar	2,489 (132)	.986	.059	.000	.004
Strict	2,589 (144)	.985	.058	.001	.001
Age 25					
Configural	1,648 (102)	.992	.067		
Metric	1,700 (111)	.992	.066	.000	.001
Scalar	1,821 (132)	.991	.062	.000	.004
Strict	1,929 (144)	.991	.061	.000	.001
Bifactor model (Model 3)					
Age 15					
Configural	811 (78)	.966	.043		
Metric	905 (99)	.966	.040	.000	.003
Scalar	1,030 (120)	.995	.039	.001	.001
Strict	1,134 (132)	.995	.039	.000	.000
Age 17					
Configural	749 (78)	.996	.041		
Metric	838 (99)	.995	.038	.001	.003
Scalar	944 (120)	.995	.037	.000	.001
Strict	1,059 (132)	.994	.037	.001	.000
Age 25					
Configural^a^	—	—	—		
Metric	572 (99)	.998	.038		
Scalar	700 (120)	.997	.038	.001	.000
Strict	796 (132)	.997	.039	.000	.001
Correlated errors (Model 4)					
Age 15					
Configural	1,638 (78)	.992	.063		
Metric	2,174 (89)	.989	.068	.003	.005
Scalar	2,536 (112)	.987	.065	.002	.003
Strict	2,657 (124)	.986	.064	.001	.001
Age 17					
Configural	1,696 (78)	.990	.064		
Metric	1,809 (89)	.989	.061	.001	.003
Scalar	2,232 (112)	.987	.061	.002	.000
Strict	2,311 (124)	.987	.059	.000	.002
Age 25					
Configural	1,177 (78)	.994	.065		
Metric	1,238 (89)	.994	.062	.000	.003
Scalar	1,458 (112)	.993	.060	.001	.002
Strict	1,580 (124)	.993	.059	.000	.001

*Note.*ΔCFI ≥ .010 and ΔRMSEA ≥ .007 indicate substantial deterioration in model fit (Neufeld et al., 2023). Models are compared with the prior model consisting of one less level of constraints. Bolded values indicate a substantial decrement in model fit. CFI = Comparative Fit Index; RMSEA = Root Mean Square Error of Approximation.

a Model did not converge, also when tested separately for males and females.

#### Three-Factor Solution (Model 2)

Model fit was good according to the CFI and acceptable according to the RMSEA at all ages. In addition, residual MI could be confirmed for all three time-points across gender (see Table 5).

#### Bifactor Model (Model 3)

The configural model between males and females did not converge, which was also the case when tested separately for both genders. The other models converged. Model fit was good across all ages, and the bifactor model had the best model fit among all tested models. Residual MI was confirmed across gender (see Table 5).

#### Correlated Errors (Model 4)

Across all ages, the CFI suggested good model fit for the one-dimensional factor solution that allowed for the covariances of errors among negatively phrased items. The RMSEA indicated acceptable fit for this factor solution across all ages. ΔCFI and ΔRMSEA pointed to residual MI across gender (see Table 5).

### Mean Differences

Table 6 depicts the standardized mean differences of the constructs across time and gender. The manifest sum scores were contrasted to the latent mean differences of the models with residual measurement invariance. Overall, no substantial differences emerged that would affect the conclusions drawn from different analysis. All one-factor models showed evidence that psychological distress significantly increased over time, yet effect sizes were negligible or small at best. Only when contrasting age 15 to age 25 did the bifactor model yield stronger differences than the sum score model, as indicated by non-overlapping confidence intervals, but with a negligible effect size. Across all one-factor models, females reported higher scores than males with medium effect sizes at ages 15 and 17 and small effect sizes at age 25. Only the correlated error latent model at age 15 showed greater gender differences than the sum score model (non-overlapping confidence intervals), with a small effect size. For the three-factor models, change in distress over time and across gender was of a similar magnitude to the one-factor models. However, most latent differences across time were greater than those for manifest scores (non-overlapping confidence intervals), but only half of these effects had a small effect size, whereas the others were negligible. For gender, all three-factor models yielded comparable findings across latent and manifest scores.

**Table 6:** Standardized Mean Differences of Manifest and Latent Scores Across Time and Gender

Model	Age 15–17	Age 15–25	Age 17–25	Gender age 15	Gender age 17	Gender age 25
One-factor solutions						
Sum score manifest	−0.04 [−0.05, −0.02]	−0.12 [−0.14, −0.10]^a^	−0.09 [−0.12, −0.05]	0.46 [0.41, 0.51]^a^	0.44 [0.39, 0.49]	0.20 [0.14, 0.26]
One-factor solution latent (Model 1)	−0.05 [−0.08, −0.02]	−0.14 [−0.18, −0.11]	−0.09 [−0.11, −0.06]	0.51 [0.45, 0.57]	0.48 [0.42, 0.54]	0.20 [0.13, 0.27]
Bifactor model general factor (Model 3)	−0.07 [−0.11, −0.03]	−0.23 [−0.27, −0.18]	−0.15 [−0.19, −0.11]	0.48 [0.42, 0.54]	0.45 [0.38, 0.51]	0.17 [0.09, 0.25]
Correlated error model latent (Model 4)	−0.06 [−0.09, −0.02]	−0.17 [−0.21, −0.12]	−0.11 [−0.15, −0.07]	0.60 [0.53, 0.67]	0.56 [0.49, 0.63]	0.21 [0.14, 0.29]
Three-factor solutions (Model 2)						
Social dysfunction manifest	−0.01 [−0.02, −0.00]	−0.11 [−0.13, −0.09]^a^	−0.11 [−0.12, −0.09]^a^	0.34 [0.29, 0.39]	0.30 [0.25, 0.35]	0.12 [0.06, 0.18]
Social dysfunction latent	−0.04 [−0.08, −0.01]	−0.23 [−0.28, −0.17]	−0.19 [−0.25, −0.14]	0.44 [0.37, 0.50]	0.39 [0.32, 0.45]	0.13 [0.06, 0.21]
Depression/Anxiety manifest	−0.10 [−0.12, −0.08]^a^	−0.16 [−0.19, −0.13]^a^	−0.06 [−0.09, −0.03]	0.44 [0.39, 0.49]	0.43 [0.38, 0.48]	0.21 [0.15, 0.27]
Depression/Anxiety latent	−0.18 [−0.24, −0.13]	−0.31 [−0.37, −0.22]	−0.13 [−0.19, −0.06]	0.48 [0.42, 0.55]	0.48 [0.42, 0.54]	0.22 [0.15, 0.30]
Confidence manifest	0.03 [0.01, 0.05]	−0.07 [−0.10, −0.04]^a^	−0.10 [−0.13, −0.07]^a^	0.38 [0.34, 0.43]	0.38 [0.33, 0.43]	0.20 [0.14, 0.27]
Confidence latent	0.07 [−0.06, 0.20]	−0.37 [−0.25, −0.51]	−0.45 [−0.57, −0.32]	0.43 [0.37, 0.50]	0.45 [0.38, 0.52]	0.28 [0.20, 0.35]

*Note.* Latent scores are based on strict measurement invariance models; gender differences refer to female–male differences. Values in brackets show 99% confidence intervals, (Cohen, 1988). Findings were equivalent with 95% confidence intervals.

a Non-overlapping confidence interval of the manifest sum score and one of the latent scoring methods.

## Discussion

In the present study, we examined MI of the GHQ-12 from adolescence into emerging adulthood and across gender in a national cohort study representative of UK. We tested the MI constraints in a simple one-factor solution (Model 1), a three-factor solution with correlated latent factors (Model 2), a bifactor model with two specific factors accounting for the positive and negative item wordings (Model 3), and a one-dimensional factor allowing for the covariance of the error terms of the negatively phrased items (Model 4).

### Factor Solutions

Essentially, all factor solutions had acceptable to good model fit over time in line with many studies indicating excellent psychometric properties of the GHQ-12 (Gnambs & Staufenbiel, 2018; Hystad & Johnsen, 2020). Recent meta-analyses (Gnambs & Staufenbiel, 2018), and other studies in adults (Hystad & Johnsen, 2020) and adolescents (Pérez et al., 2020) have concluded that the GHQ-12 is essentially unidimensional. Although this model may be the most relevant solution for applied research, our findings support the importance of the two factors capturing variance related to item wording. Accounting for this variance by either using the bifactor model or allowing for the covariances of errors led to improved model fit. Descriptively, the bifactor model (Model 3) had the best model fit over time. However, as bifactor models tend to overfit data, goodness-of-fit statistics alone should not be used to support this model (Bonifay & Cai, 2017). Although most variances in the bifactor model were attributable to the overarching latent factor (69%–75%), this was not at a level that would support construing the GHQ as solely a unidimensional construct (Rodriguez et al., 2016). In addition, model fit improved descriptively when allowing for error covariances of negatively formulated items (Model 4). Former studies have concluded that the response options of the negatively worded items may be ambiguous or that negatively worded items evoke slightly different response patterns (Hankins, 2008; Rey et al., 2014). Our findings suggest that the interpretation of negatively worded items may be especially relevant in research in adolescents compared to emerging adults, as the overarching latent factor explained more variance at age 25 compared to ages 15 and 17. However, the specific factors from the bifactor model may capture more than method variance and may represent substantive factors with their own meaning. Future research should test whether these factors contribute uniquely to important outcome measures. This would align with the three-factor model, which conceptualizes them as substantive correlated factors. This model had good model fit, which is in line with previous studies (Abubakar & Fischer, 2012; French & Tait, 2004; Graetz, 1991). However, the utility of the three-factor solution has been questioned given that it also splits positive and negative items and provides little meaningful information beyond simple GHQ-12 scores (Shevlin & Adamson, 2005). One additional caveat of the three-factor model is that the confidence factor consists of two indicators and is thus only identified via its correlations with the other factors. This can lead to unstable factor loading estimates.

### Measurement Invariance

The highest level of MI across time and gender was mainly established for all factor solutions. This extends the scope of previous studies that have tested MI of different GHQ-12 factor solutions in adults across clinical and non-clinical populations (Fernandes & Vasconcelos-Raposo, 2013), ethnic groups (Bowe, 2017; King et al., 2023), cultures (Romppel et al., 2017), men and women (Shevlin & Adamson, 2005), time (Mäkikangas et al., 2006), and before and during COVID-19 (Schlechter, et al., 2023). This demonstration of MI supports the usage of the GHQ-12 in adolescents and emerging adults, as mean differences across time or gender seem to reflect true-score differences on the latent variables (Liu et al., 2017). This is critical for a measurement tool as widely applied as the GHQ-12 (Gnambs & Staufenbiel, 2018). Despite the ongoing psychological, social, and biological changes occurring during this developmental phase (Dahl et al., 2018), the GHQ-12 appears applicable to discern differences in distress over time and gender from adolescence into emerging adulthood. However, this only accounts for the age range that we investigated here, and independent testing for younger ages is necessary. Specifically, in previous research, the Short Mood and Feelings Questionnaire (SMFQ) demonstrated longitudinal MI from age 14 to 26 but not when ages 11–13 were included (Schlechter et al., 2023). In addition, the Social-Behavior-Questionnaire functioned psychometrically well across ages 11 to 17 but displayed minor violations of MI in younger ages (mainly ages 11 and 13; Murray et al., 2019). The only deviation from MI was found for the bifactor model (Model 3) when transitioning from the metric to the scalar invariance model. However, the deviation was only found according to the RMSEA but not according to the CFI. Moreover, overall model fit was still good for this model and also for the residual MI model. We refrained from testing partial invariance for this model because testing partial MI is sample-dependent, exploratory, and does not contribute to the general applicability of the GHQ-12 (Neufeld et al., 2023). However, we observed that thresholds were lower at age 25 than at other ages. This accounted for different items such as “restless sleep” or “being under strain.” At age 25, emerging adults experience more life stressors (Arnett, 2000), which may influence their reporting of these symptoms.

Also, when testing the one-factor solution (Model 1) cross-sectionally at each wave for gender, model fit was only good according to the CFI but not the RMSEA. However, this was likely attributable to the discussed method effects of the negative phrasing of the items (Hankins, 2008; Rey et al., 2014) because there were no problems in the models accounting for these effects (Models 3 and 4). Moreover, for all models that were tested, we drew the same conclusions about gender differences between females and males.

### Mean Differences

Although sum score models did not unequivocally fit the data well, mean differences across gender and time did not strongly deviate from each other regardless of whether sum scores or latent difference scores were used. In addition, reliability estimates were good and extremely similar regardless of whether omega total (taking different factor loadings into account) or Cronbach alphas (assuming equal factor loadings) were used (Widaman & Revelle, 2023). This points to the robustness of the GHQ-12 scoring, which is important given that the use of sum scores is based on strict assumptions (McNeish & Wolf, 2020; Widaman & Revelle, 2023).

In all models, psychological distress increased in young people over time, in line with epidemiological studies (Solmi et al., 2022), although effect sizes were generally no bigger than small. Females reported higher levels of psychological distress than males with generally small-to-moderate effect sizes, also consistent with previous work reporting, for instance, higher levels of depression among females and a steeper increase at younger ages (Hyde & Mezulis, 2020; Patalay & Fitzsimons, 2018; Salk et al., 2017; Shore et al., 2018; Solmi et al., 2022). The present work helpfully clarifies that such gender differences do not contribute to lack of MI in the GHQ but are reflected in genuine differences in the latent distress construct.

In some instances, especially for the three-factor model over time, differences were greater for latent scores than for manifest scores, potentially because of fewer items contributing to the single scores in this factor solution, leaving more room for effects of measurement error. This highlights the importance of using latent scores when constructs are assessed with only a few items, to ensure accuracy in observed differences.

### Limitations

Attrition over time is a limitation of our study. Although variables that were associated with missingness were identified, unmeasured variables may have influenced attrition (Graham, 2009). Parameter estimates of models are only unbiased under the assumption of MAR (White et al., 2011). However, sensitivity analyses using multiple imputation supported our MI findings as there was no deviation in the findings. Thus far, there is also no consensus on how to establish MI across time with ordinal data (see Liu et al., 2017; Neufeld et al., 2023). Using the χ^2^ test statistics or difference tests may lead to inflated Type 1 error rates, especially with large sample sizes as in the present analyses. Changes in fit indices have not yet been conclusively examined (Liu et al., 2017). Moreover, it has been argued that constraints on factor loadings and item thresholds should be investigated simultaneously in one model when ordinal data are used (Chen et al., 2020). This is because they jointly influence participants’ responses to a given response option. However, for a more fine-grained analysis, we tested the steps of metric and scalar MI separately (Millsap & Yun-Tein, 2004). In addition, it would be ideal to have data with more regular GHQ assessments throughout adolescence and emerging adulthood. Although unlikely, it is possible that non-linear developmental changes in distress symptoms after age 17 and before age 25 could mean the GHQ is not fully invariant during this time. However, in our prior study on MI in adolescent depressive symptoms, which had many items common to the GHQ (covering concentration, tiredness, enjoying activities, feeling unhappy and worthless), strict invariance was established from ages 14 to 26, including seven waves from ages 17 to 25 (Schlechter et al., 2023). This supports the notion that GHQ is invariant during this time, although future datasets could more definitively clarify this. Finally, gender was assessed with a single question, which is common for cohort studies that started many years ago. Therefore, we cannot distinguish between biological sex and gender identity. Future research should be more inclusive and examine MI across broad gender categorizations (Richards et al., 2016).

### Conclusion

Using longitudinal representative UK data of young people of ages 15 to 25 years, the present study contributes further knowledge to MI of the GHQ-12. For all factor solutions, meaningful comparisons of psychological distress across time and gender seem justified. While factor solutions accounting for the effects of the item wording yielded better model fit than a simple unidimensional solution, the GHQ-12 seems to essentially measure one construct of psychological distress. Findings indicate that practitioners and researchers can confidently use GHQ to assess how psychological distress unfolds and pervades during the sensitive period of adolescence and emerging adulthood.

## Supplemental Material

sj-docx-1-asm-10.1177_10731911241229573 – Supplemental material for Longitudinal and Gender Measurement Invariance of the General Health Questionnaire-12 (GHQ-12) From Adolescence to Emerging Adulthood Supplemental material, sj-docx-1-asm-10.1177_10731911241229573 for Longitudinal and Gender Measurement Invariance of the General Health Questionnaire-12 (GHQ-12) From Adolescence to Emerging Adulthood by Pascal Schlechter and Sharon A. S. Neufeld in Assessment
