# Supplementary material for: Longitudinal and Gender Measurement Invariance of the General Health Questionnaire-12 (GHQ-12) From Adolescence to Emerging Adulthood
Source: Assessment. 2024 Feb 12;31(8):1687–701. doi: 10.1177/10731911241229573 (PMC11492552; doi:10.1177/10731911241229573)
Supplement: sj-docx-1-asm-10.1177_10731911241229573 – Supplemental material for Longitudinal and Gender Measurement Invariance of the General Health Questionnaire-12 (GHQ-12) From Adolescence to Emerging Adulthood [file sj-docx-1-asm-10.1177_10731911241229573.docx]

| **Table s1.** Missingness in GHQ-12 scores | |  |  |  |
| --- | --- | --- | --- | --- |
|  | Data available | | No data available | *p* |
| **Age 15** |  | |  |  |
| Gender |  | |  |  |
| Male | 5304 | | 1436 | ˂.001 |
| Female | 4820 | | 1757 |  |
| Ethnicity MP age 14 |  | |  |  |
| White | 7360 | | 2213 | ˂.001 |
| Mixed | 154 | | 58 |  |
| Indian | 647 | | 246 |  |
| Pakistani | 543 | | 289 |  |
| Bangladeshi | 400 | | 233 |  |
| Black Caribbean | 369 | | 140 |  |
| Black African | 342 | | 120 |  |
| Other | 237 | | 84 |  |
| Family income age 14 |  | |  |  |
| £ | 15310 | | 12959 | ˂.001 |
| University education MP |  | |  |  |
| Yes | 332 | | 51 | ˂.001 |
| No | 9673 | | 3303 |  |
| Employment MP |  | |  |  |
| Full-time paid employee | 4404 | | 1166 | ˂.001 |
| Part-time paid employee | 487 | | 142 |  |
| Full-time self-employed | 767 | | 199 |  |
| Part-time self-employed | 54 | | 14 |  |
| Unemployed/ seeking work | 146 | | 95 |  |
| Full-time education | 18 | | 9 |  |
| Employment training | 4 | | 2 |  |
| Temporarily sick/ disabled | 52 | | 20 |  |
| Permanently sick/ disabled | 192 | | 70 |  |
| Looking after home/ family | 546 | | 283 |  |
| Retired from work | 71 | | 29 |  |
| Other | 21 | | 10 |  |
| **Age 17** |  | |  |  |
| Gender |  | |  |  |
| Male | 5187 | | 1553 | .99 |
| Female | 5062 | | 1515 |  |
| Ethnicity MP age 14 |  | |  |  |
| White | 7534 | | 2039 | ˂.001 |
| Mixed | 144 | | 68 |  |
| Indian | 698 | | 195 |  |
| Pakistani | 585 | | 247 |  |
| Bangladeshi | 440 | | 193 |  |
| Black Caribbean | 340 | | 169 |  |
| Black African | 292 | | 170 |  |
| Other | 220 | | 101 |  |
| Annual salary MP 14 |  | |  |  |
| £ | 14129 | | 14946 | .074 |
| University education MP |  | |  |  |
| Yes | 311 | | 72 | .032 |
| No | 9907 | | 3069 |  |
| Employment MP |  | |  |  |
| Full-time paid employee | 4532 | | 1038 | ˂.001 |
| Part-time paid employee | 495 | | 134 |  |
| Full-time self-employed | 776 | | 190 |  |
| Part-time self-employed | 57 | | 11 |  |
| Unemployed/ seeking work | 163 | | 78 |  |
| Full-time education | 20 | | 7 |  |
| Employment training | 5 | | 1 |  |
| Temporarily sick/ disabled | 56 | | 16 |  |
| Permanently sick/ disabled | 191 | | 71 |  |
| Looking after home/ family | 592 | | 237 |  |
| Retired from work | 78 | | 22 |  |
| Other | 20 | | 11 |  |
| **Age 18** |  | |  |  |
| Gender |  | |  |  |
| Male | 2954 | | 3786 | ˂.001 |
| Female | 3712 | | 2865 |  |
| Ethnicity MP age 14 |  | |  |  |
| White | 4950 | | 4623 |  |
| Mixed | 84 | | 128 |  |
| Indian | 468 | | 425 |  |
| Pakistani | 375 | | 457 |  |
| Bangladeshi | 305 | | 328 |  |
| Black Caribbean | 200 | | 309 |  |
| Black African | 174 | | 288 |  |
| Other | 134 | | 187 |  |
| Family income age 14 |  | |  |  |
| £ | 14285 | | 15204 | .017 |
| University education MP |  | |  |  |
| Yes | 212 | | 171 | .035 |
| No | 6456 | | 6520 |  |
| Employment MP |  | |  |  |
| Full-time paid employee | 3067 | | 2503 | ˂.001 |
| Part-time paid employee | 345 | | 284 |  |
| Full-time self-employed | 506 | | 460 |  |
| Part-time self-employed | 37 | | 31 |  |
| Unemployed/ seeking work | 103 | | 138 |  |
| Full-time education | 12 | | 15 |  |
| Employment training | 3 | | 3 |  |
| Temporarily sick/ disabled | 36 | | 36 |  |
| Permanently sick/ disabled | 138 | | 124 |  |
| Looking after home/ family | 387 | | 442 |  |
| Retired from work | 46 | | 54 |  |
| Other | 16 | | 15 |  |

|  | **One-factor (Model 1)** | | | | | | | **Three-factors (Model 2)** | | | | | |  |  |
| --- | --- | --- | --- | --- | --- | --- | --- | --- | --- | --- | --- | --- | --- | --- | --- |
|  | $\chi^{2}(df)$ | CFI | | RMSEA | ∆CFI | | ∆RMSEA | $\chi^{2}(df)$ | | CFI | RMSEA | ∆CFI | ∆RMSEA |  |  |
| Configural | 31425 (555) | .965 | | .065 |  | |  | 10908 (522) | | .988 | .039 |  |  |  |  |
| Metric | 33949 (577) | .962 | | .066 | .003 | | .001 | 13164 (540) | | .986 | .042 | .002 | .003 |  |  |
| Scalar | 41508 (613) | .954 | | .071 | .008 | | .005 | 19236 (576) | | .979 | .049 | .007 | .007 |  |  |
| Strict | 43884 (649) | .951 | | .071 | .003 | | .000 | 23058 (612) | | .975 | .050 | .004 | .001 |  |  |
| Sum | 82827 (588) | .907 | | .102 | **.058** | | **.037** | 21443 (549) | | .976 | .053 | **.021** | **.014** |  |  |
|  |  |  | **Bifactor (Model 3)** | | |  | | | **Correlated errors (Model 4)** | | | | | | |
| Configural | 6120 (513) | .994 | | .029 |  | |  | 20297 (510) | | .978 | .054 |  |  |  |  |
| Metric | 8123 (555) | .991 | | .032 | .003 | | .003 | 21895 (532) | | .976 | .055 | .002 | .001 |  |  |
| Scalar | 16184 (591) | .982 | | .045 | .009 | | **.012** | 29742 (568) | | .967 | .062 | .009 | .007 |  |  |
| Strict | 18145 (627) | .980 | | .046 | .002 | | .001 | 32149 (604) | | .964 | .063 | .003 | .001 |  |  |

**Table s2.** Longitudinal measurement invariance models using 10 imputed data sets.

Note. CFI = Comparative Fit-Index; RMSEA = Root Mean Square Error of Approximation; *df* = degrees of freedom,

ΔCFI ≥ .010 and ΔRMSEA ≥ .007 indicate substantial deterioration in model fit (Neufeld et al., 2022). Models are compared with the prior model consisting of one less level of constraints.

Adequate pooling procedures for these fit indices across multiple imputed data sets have not yet been established (Liu et al., 2017). We therefore used naïve averages of these fit indices across imputations to evaluate model fit.

**Bolded** values indicate a substantial decrement in model fit.

| **Table s3.** Measurement invariance across gender using 10 imputed data sets. | | | | | |
| --- | --- | --- | --- | --- | --- |
|  | $\chi^{2}(df)$ | CFI | RMSEA | ∆CFI | ∆RMSEA |
| **One-factor solution (Model 1)**  **Age 15** | | | | | |
| Configural | 10274 (108) | .957 | .119 |  |  |
| Metric | 10524 (119) | .956 | .115 | .001 | .004 |
| Scalar | 10553 (142) | .956 | .105 | .000 | **.010** |
| Strict | 10718 (154) | .955 | .102 | .001 | .003 |
| **Age 17** |  |  |  |  |  |
| Configural | 7421 (108) | .964 | .101 |  |  |
| Metric | 7477 (119) | .963 | .096 | .001 | .005 |
| Scalar | 7653 (142) | .963 | .089 | .000 | **.007** |
| Strict | 7724 (154) | .962 | .086 | .001 | .003 |
| **Age 25** |  |  |  |  |  |
| Configural | 8505 (108) | .972 | .108 |  |  |
| Metric | 8578 (119) | .971 | .103 | .001 | .005 |
| Scalar | 8675 (142) | .971 | .095 | .000 | **.008** |
| Strict | 8772 (154) | .971 | .092 | .000 | .003 |
| **Three-factor solution (Model 2)**  **Age 15** | | | | | |
| Configural | 2958 (102) | .988 | .065 |  |  |
| Metric | 3083 (111) | .987 | .063 | .001 | .002 |
| Scalar | 3212 (132) | .987 | .059 | .000 | .004 |
| Strict | 3313 (144) | .987 | .057 | .000 | .002 |
| **Age 17** |  |  |  |  |  |
| Configural | 2581 (102) | .986 | .064 |  |  |
| Metric | 2885 (111) | .986 | .061 | .000 | .003 |
| Scalar | 2992 (132) | .986 | .057 | .000 | .004 |
| Strict | 3084 (144) | .985 | .055 | .001 | .002 |
| **Age 25** |  |  |  |  |  |
| Configural | 3005 (102) | .990 | .065 |  |  |
| Metric | 3048 (111) | .990 | .063 | .000 | .002 |
| Scalar | 3142 (132) | .990 | .059 | .000 | .004 |
| Strict | 3215 (144) | .990 | .057 | .000 | .002 |
| **Bifactor model (Model 3)**  **Age 15** | | | | | |
| Configural | 1049 (78) | .996 | .043 |  |  |
| Metric | 1102 (99) | .996 | .039 | .000 | .004 |
| Scalar | 1269 (120) | .995 | .038 | .001 | .001 |
| Strict | 1352 (132) | .995 | .037 | .000 | .001 |
| **Age 17** |  |  |  |  |  |
| Configural | 1025 (78) | .995 | .043 |  |  |
| Metric | 1108 (99) | .995 | .039 | .000 | .003 |
| Scalar | 1211 (120) | .995 | .037 | .000 | .002 |
| Strict | 1318 (132) | .994 | .037 | .001 | .000 |
| **Age 25** |  |  |  |  |  |
| Configural | 935 (78) | .997 | .041 |  |  |
| Metric | 1008 (99) | .997 | .037 | .000 | .004 |
| Scalar | 1102 (120) | .997 | .035 | .000 | .002 |
| Strict | 1166 (132) | .997 | .034 | .000 | .001 |
| **Correlated errors (Model 4)**  **Age 15** | | | | | |
| Configural | 2156 (78) | .991 | .063 |  |  |
| Metric | 2683 (89) | .989 | .066 | .002 | .003 |
| Scalar | 3193 (112) | .987 | .064 | .002 | .002 |
| Strict | 3273 (124) | .987 | .062 | .000 | .002 |
| **Age 17** |  |  |  |  |  |
| Configural | 2094 (78) | .990 | .062 |  |  |
| Metric | 2183 (89) | .990 | .059 | .000 | .003 |
| Scalar | 2638 (112) | .987 | .058 | .003 | .001 |
| Strict | 2712 (124) | .987 | .056 | .000 | .002 |
| **Age 25** |  |  |  |  |  |
| Configural | 2072 (78) | .993 | .062 |  |  |
| Metric | 2125 (89) | .993 | .059 | .000 | .003 |
| Scalar | 2327 (112) | .993 | .055 | .000 | .004 |
| Strict | 2397 (124) | .992 | .052 | .001 | .003 |

*Note.* Adequate pooling procedures for these fit indices across multiple imputed data sets have not yet been established (Liu et al., 2017). We therefore used naïve averages of these fit indices across imputations to evaluate model fit.

ΔCFI ≥ .010 and ΔRMSEA ≥ .007 indicate substantial deterioration in model fit (Neufeld et al., 2022) . Models are compared with the prior model consisting of one less level of constraints. **Bolded** values indicate a substantial decrement in model fit.
